# The Czech Surveillance System for Invasive Pneumococcal Disease, 2008-2013: A Follow-Up Assessment and Sensitivity Estimation

**DOI:** 10.1371/journal.pone.0131117

**Published:** 2015-06-30

**Authors:** Nina Katharina Stock, Marek Maly, Helena Sebestova, Hana Orlikova, Jana Kozakova, Pavla Krizova

**Affiliations:** 1 National Institute of Public Health (NIPH), Prague, Czech Republic; 2 European Program for Public Health Microbiology (EUPHEM), ECDC, Stockholm, Sweden; Faculdade de Medicina de Lisboa, PORTUGAL

## Abstract

**Background:**

Invasive pneumococcal disease (IPD) is caused by *Streptococcus pneumoniae* and mostly presents as pneumonia, sepsis or meningitis. A notable portion of IPD cases is vaccine preventable and the pneumococcal conjugate vaccine (PCV) was introduced into the routine childhood immunization programs in many countries during the last decades.

**Objectives:**

Before PCV introduction in the Czech Republic in 2010, a national surveillance system for IPD was implemented in 2008 and further improved in 2011. In this study, we describe the new surveillance system for the first time and measure its sensitivity between 2010 and 2013 using the capture-recapture method. Furthermore, we describe the recent epidemiological trend of IPD, taking sensitivity estimates into account.

**Results and Conclusions:**

Between 2010 and 2013 the estimated sensitivity of the overall IPD surveillance increased from 81% to 99%. The sensitivity of individual reporting sources increased from 72% to 87% for the laboratory system and from 31% to 89% for the epidemiological notification system. Crucial for this improvement was the introduction of quarterly report reminders in 2011. Due to positive source dependency, the presented sensitivity estimates are most probably overestimated and reflect the upper limit of reporting completeness. Stratification showed variation in sensitivity of reporting particularly according to region.An effect of the PVC vaccination in the Czech Republic is visible in the incidence of IPD in target age groups (<5y). This influence was not evident in the total IPD incidence and may interfere with increasing sensitivity of reporting. In 2013, an increase in the IPD incidence was observed. This finding requires further observation and a detailed vaccine impact analysis is needed to assess the current immunization strategy.

## Introduction

*Streptococcus pneumoniae* can be found in the nasopharyngeal flora of healthy humans and commonly causes non-invasive infections such as otitis media or sinusitis. Invasion of *S*. *pneumoniae* to normally sterile body sites leads to invasive pneumococcal disease (IPD), causing high morbidity and mortality worldwide mainly in children and the elderly. Humans are the only host and transmission occurs through respiratory droplets of infected or colonized individuals [1–3]. The clinical presentation of IPD can be diverse, but commonly presents as pneumonia, sepsis or meningitis. The 2008 European IPD case definition requires laboratory confirmation of *S*. *pneumoniae* from normally sterile body sites such as cerebrospinal fluid, blood or pleural fluid, identified by bacterial culture, PCR or antigen detection [3]. Based on the nature of capsular polysaccharides, 94 different serotypes have been identified thus far [4]. Prevention of infection with certain serotypes is achieved with two types of vaccines: the pneumococcal polysaccharide vaccine PPV23 and the pneumococcal conjugate vaccines PCV7, PCV10 and PCV13. PCVs have been licensed since 2001 in the EU and have been implemented into the routine childhood immunization of many countries worldwide within the last decades [2–6].

**Fig 1:**
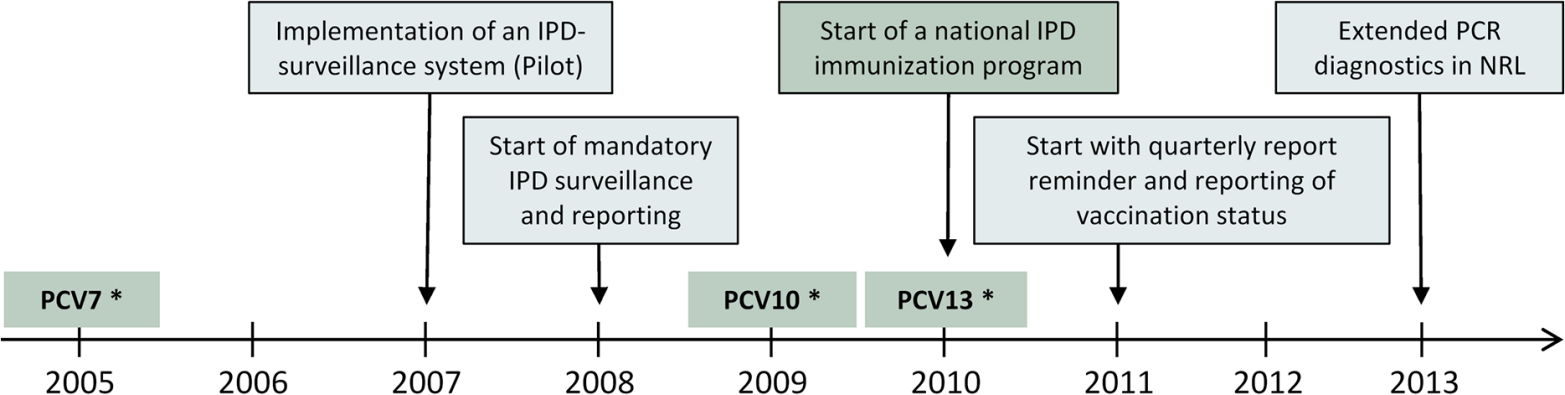
Timeline of events related to the introduction of PCV vaccination and IPD surveillance in the Czech Republic, 2005–2013. * Licensed and available on the private market.

In the Czech Republic (CZ), the first PCV (PCV7) was registered in 2005 and was available for immunization in the private market (Fig 1). With respect to the registration of PCV10 (2009) and PCV13 (2010), a comprehensive national surveillance system for IPD was piloted in 2007, replacing the non-compulsory laboratory system described previously [7,8]. Since 2008 IPD reporting is mandatory and in 2010 vaccination with PCV10 and PCV13 was included in the Czech national childhood immunization program, following an immunization schedule of 3+1 at 2, 3, 4 and 12 months of age (Fig 1).

European IPD surveillance was previously evaluated in two independent, international surveys, one conducted between 2003 and 2006 [8] and the other in 2010 [5]. These surveys showed an improvement in surveillance activities over time. Nevertheless it was underlined that IPD surveillance still remains very heterogeneous between EU countries, leading to difficulties in comparing epidemiological data. In 2011 for example, the overall IPD incidence in the EU/EEA was 5.6 per 100.000, ranging from 0.3 in Lithuania to 16.6 in Denmark [9]. Differences in surveillance data might reflect the application of different case definitions, different reporting systems, vaccination programs, as well as differences in medical and diagnostic practices such as blood culturing habits [5].

The need for comparable European surveillance data was highlighted with regard to monitoring incidence over time, recording potential serotype replacement as well as analysing the impact of specific vaccination programs as an important basis for decision making on vaccination strategies and disease management. Therefore, a well-structured and comprehensive surveillance of IPD is essential and regular sensitivity assessments of individual surveillance systems would be of considerable value as additional information to reflect correct incidence rates [5,10].

In order to improve the pan-European IPD surveillance and to evaluate the impact of PCV vaccination in the EU, a pilot project that aimed at setting up active population based IPD surveillance was initiated in 2012 in eight EU/EEA countries, including the CZ (ECDC funded SpIDnet project) [11]. The Czech country-specific action plan included the continuous improvement of the surveillance system, the evaluation of reporting sensitivity as well as the analysis of vaccine effectiveness and impact of the recently introduced PCV vaccination.

In the present study we describe the newly implemented Czech IPD surveillance system since 2008 and measure sensitivity of reporting from 2010 to 2013 using the capture-recapture method. Sensitivity estimates are considered in the discussion of the IPD epidemiological trend in the CZ.

## Methods

### IPD surveillance 2008–2013: case definition, reporting and data selection

**Fig 2:**
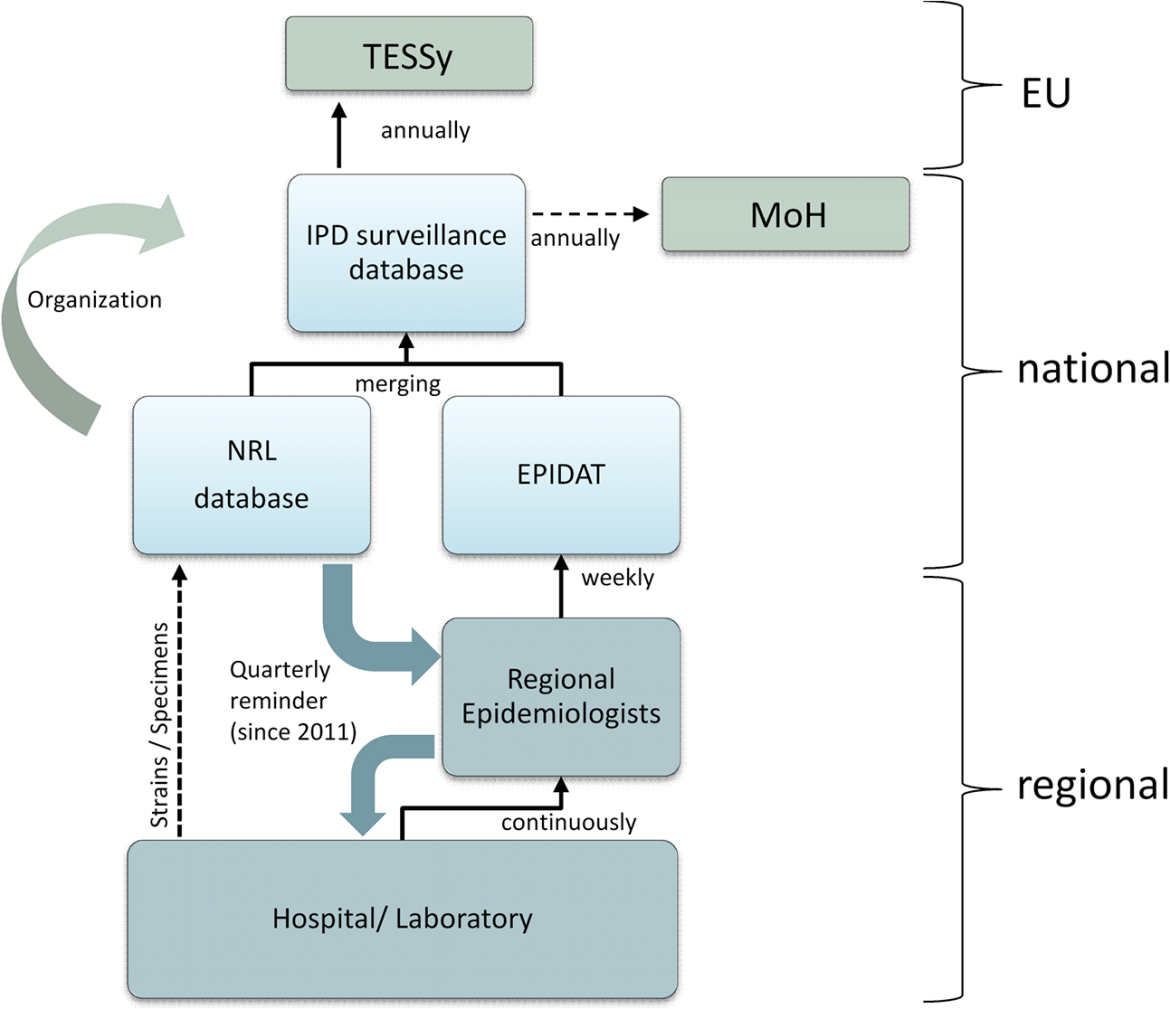
Structure and levels of the Czech IPD surveillance system, based on two reporting sources. NRL = National Reference Laboratory for streptococcal infections, EPIDAT = Czech national epidemiological database, MoH = Ministry of Health, TESSy = The European Surveillance System.

The population under surveillance is the whole Czech population, using annual census population data as the denominator (source: www.czso.cz). IPD cases are reported in a passive stimulated manner according to the 2008 EU case definition to two different notification systems (Fig 2).

The first way of reporting is undertaken by clinicians and field laboratories to regional epidemiologists, who further investigate and complete data related to individual cases. Complete cases are reported weekly to the National Reference Centre of Analysis of Epidemiological Data (National Institute of Public Health, Prague) using an electronic epidemiological database (EPIDAT), which is part of the National Health Information system. IPD cases reported to EPIDAT are diagnosed by culture, PCR or antigen detection and reporting occurs using codes for international classification of diseases (ICD10). Due to the diverse clinical presentation of the disease, IPD reporting is not uniform with regard to code utilisation. For case ascertainment, data are screened for cases reported with ICD codes A40 (streptococcal sepsis), A41 (other sepsis), G00 (bacterial meningitis) and J17 (pneumonia) together with the string variable ‘*Strep*. *pneumoniae*’. Cases consciously identified from non-sterile site specimens are excluded as not meeting the case definition.

The second reporting system is via isolated strains or clinical specimens from IPD infections that are sent from field laboratories to the National reference laboratory for Streptococcal infections (NRL) for confirmation and serotyping. Cases reported to the NRL before 2013 are based on culture positive isolates only. Since 2013, the NRL also performs PCR testing from culture negative clinical specimens and extracted DNA.

Data retrieved from both sources are merged with the exclusion of duplicates using unique personal identifiers attributed to each Czech resident based on date of birth, sex and individual numeric codes, to produce a final consolidated dataset (IPD surveillance database). IPD surveillance is organised and maintained at the NRL. In 2011, the surveillance system was further improved by including the report of the vaccination status of IPD cases and implementing quarterly report reminders sent from the NRL to regional epidemiologists. This reminder retrospectively stimulates the reporting of missing cases to the EPIDAT system (Figs 1 and 2).

Cumulative data are reported further to the Ministry of Health (MoH) and case-based data to the European Surveillance System (TESSy) annually. Case-based reporting includes data on demographics, hospitalization, diagnosis, specimen and outcome.

### Statistical methods

#### Capture-recapture and sensitivity calculation

In order to estimate the true number of IPD cases in the Czech population, we applied the capture-recapture method with two sources [12–14]. A Poisson log-linear model was used to estimate the total number of cases and to test the differences between subgroups or years [15]. Model parameters were estimated using a maximum likelihood approach. Corresponding 95% confidence intervals were calculated for each estimate using profile likelihood methods [16].

We calculated the sensitivity of reporting as the percentage of reported cases relative to the estimated true number of cases according to the formula Se(%) = N/NE x 100; where Se = estimated sensitivity, N = number of reported cases, NE = estimated true number of cases calculated by capture-recapture.

The significance level was set to 0.05. The analysis was performed with Stata, release 9.2 (Stata Corp LP, College Station, U.S.A.) and R [17].

#### Estimation of source dependency

The presence and the extent of possible source dependency between the two data sources was estimated for all years as described by Hook et al., where relative odds ratios (OR) >1 reflect positive source dependency and OR<1 indicate negative source dependency [14]. Source dependency was further estimated by applying a method by Sekar and Deming [18], which describes underestimation of the estimated true number of cases when positive weighted correlation over strata between the numbers of missed cases in each source appears. Source independency can be assumed when the correlation coefficient does not differ significantly from 0.

#### Stratification

In order to adjust for inequality of capture probability, surveillance data from 2013 were examplary stratified by sex (male/female), disease outcome (dead/alive), age group (0-4/5-19/20-39/40-64/>65) and region (eight regions according to the Nomenclature of Units for Territorial Statistics (NUTS 2); http://epp.eurostat.ec.europa.eu/portal/page/portal/nuts_nomenclature/correspondence_tables/national_structures_eu). The estimates of reporting sensitivity were calculated for each of the defined strata as described above.

## Results

**Fig 3:**
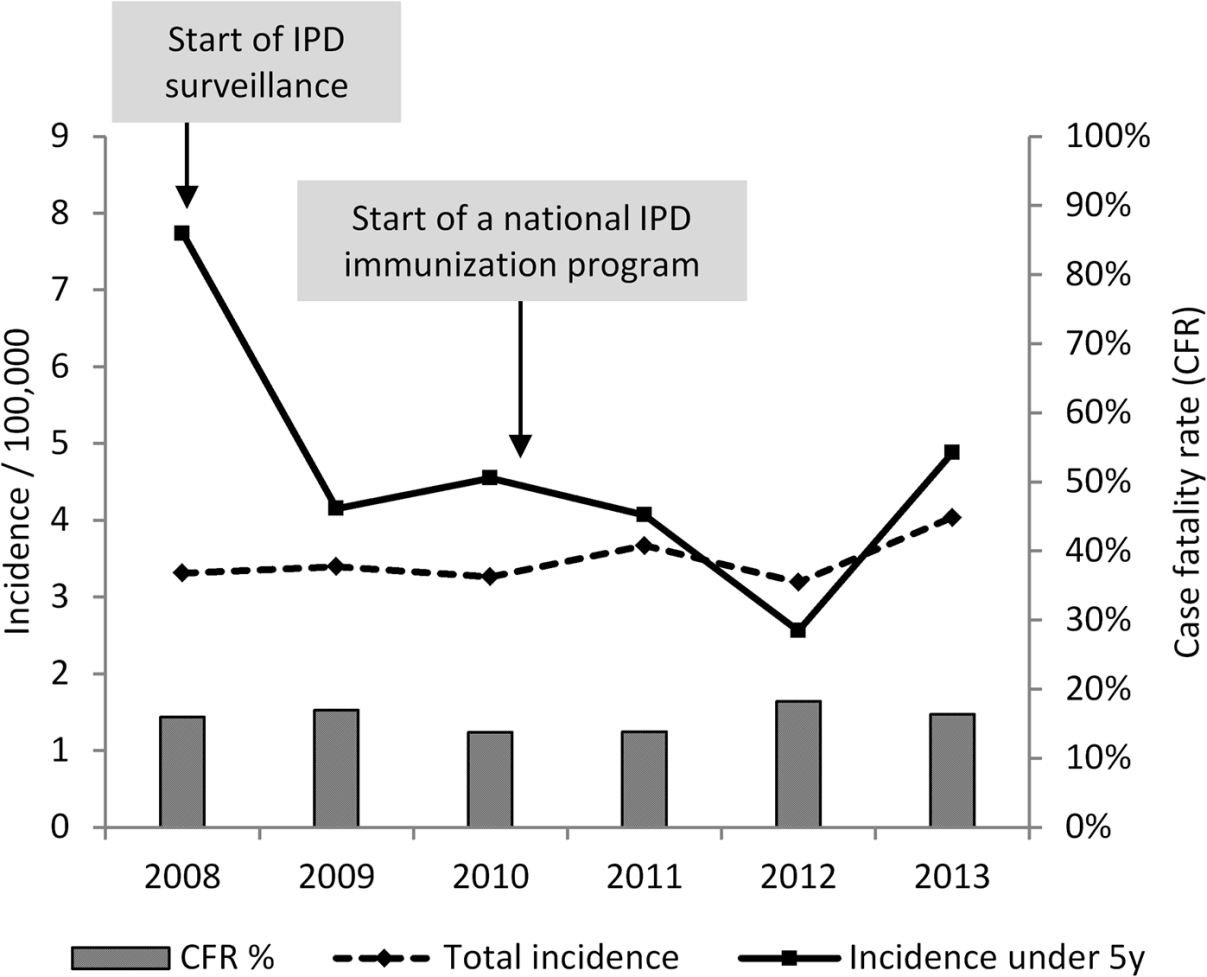
Czech annual IPD incidence in total and for children under five years (2008–2013). Bar charts reflect the total annual case fatality rate (CFR).

The total annual reported IPD incidence per 100.000 inhabitants in CZ fluctuated between 3.2 and 4.0 since 2008, with a slight increasing trend towards 2013. The age-specific incidence for children under five declined from 7.7 in 2008 to 2.6 in 2012 and increased again to 4.9 in 2013. The overall reported case fatality rates (CFR) varied between 13.7% and 18.2% in the years 2008–2013 (Fig 3).

**Fig 4:**
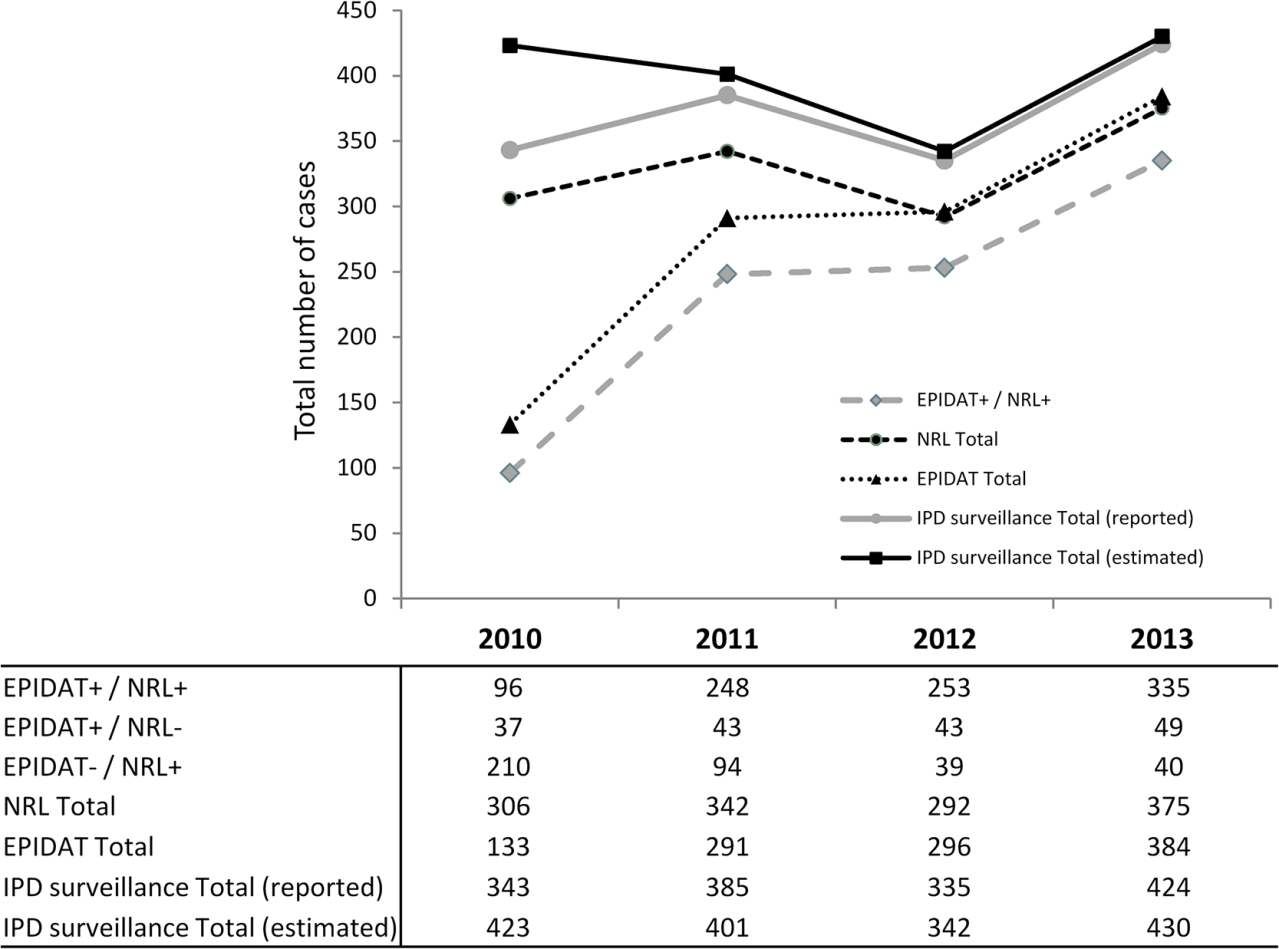
Number of reported and estimated IPD cases in the CZ according to reporting source (EPIDAT, NRL) from 2010 to 2013. + = captured by this source; - = not captured by this source.

Apart from a decrease in 2012, an overall increase in the total number of reported IPD cases was notified from 2010 to 2013, raising from 343 to 424 cases per year (24% increase). The same trend was observed in cases reported to the NRL database (306 cases in 2010 and 375 in 2013, 23% increase). The number of IPD cases reported to EPIDAT increased continuously from 133 in the year 2010 to 384 cases in 2013 (188% increase). The estimated true number of IPD cases by capture-recapture was comparable in 2010 (423), 2011 (401) and 2013 (430), but lower in 2012 (342 estimated cases) (Fig 4).

**Fig 5:**
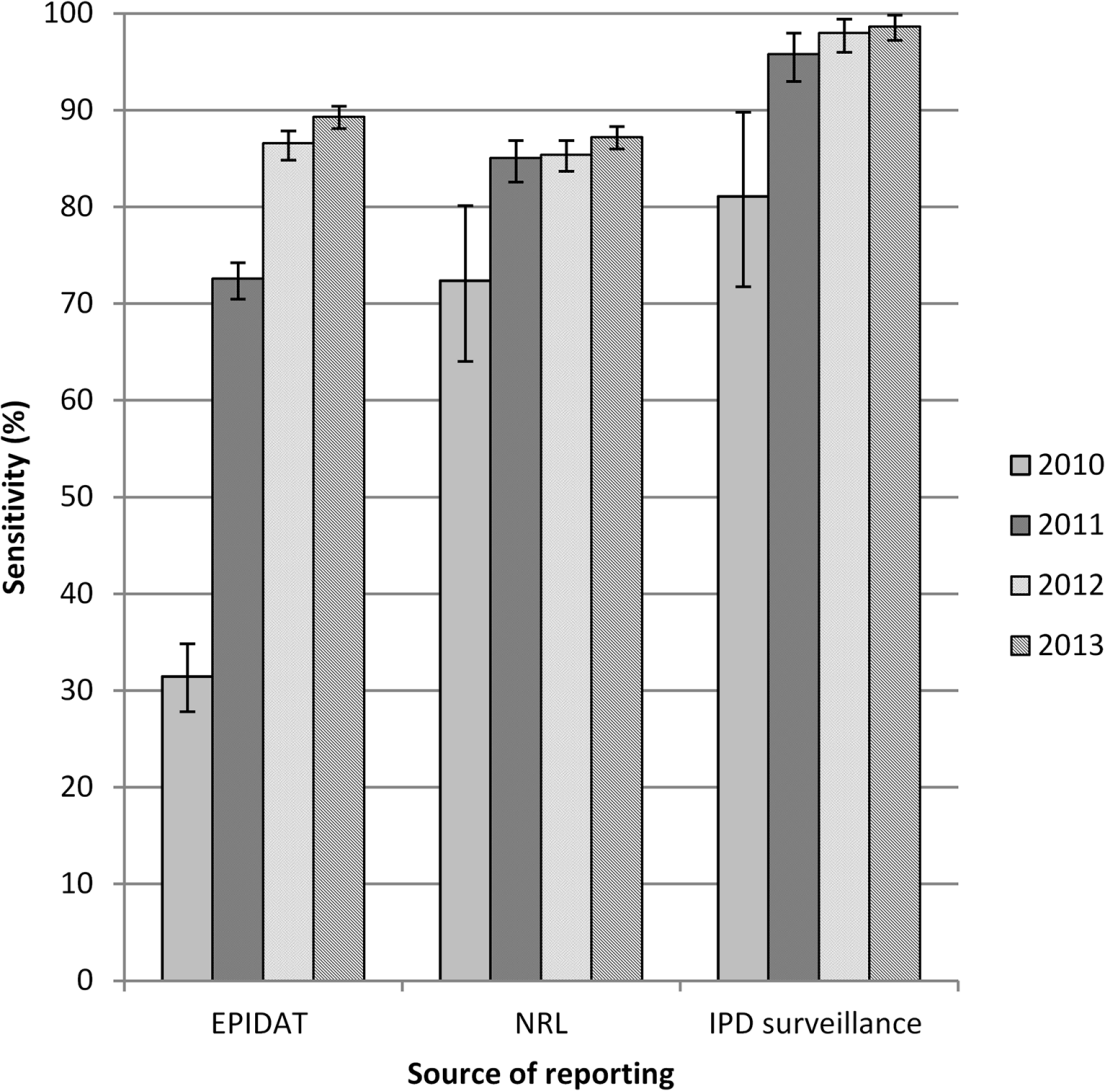
Estimated reporting sensitivity of the Czech IPD surveillance system by source of reporting and in total, 2010 to 2013. Error bars reflect 95% confidence intervals.

The overall estimated sensitivity of IPD reporting increased from 81% in 2010 to 99% in 2013 (p<0.001). The sensitivity of reporting to the NRL database alone increased from 72% to 87%, the sensitivity of the EPIDAT system from 31% to 89% in the same period of time (p<0.001 in both cases) (Fig 5).

Stratification of surveillance data from 2013 demonstrated comparable reporting sensitivity between IPD cases with fatal outcome (100%) and surviving cases (99%), and a slightly higher sensitivity in male (100%) then in female (98%). Sensitivity of reporting by age group varied between 97% (0-4y) and 100% (5-19y).

**Fig 6:**
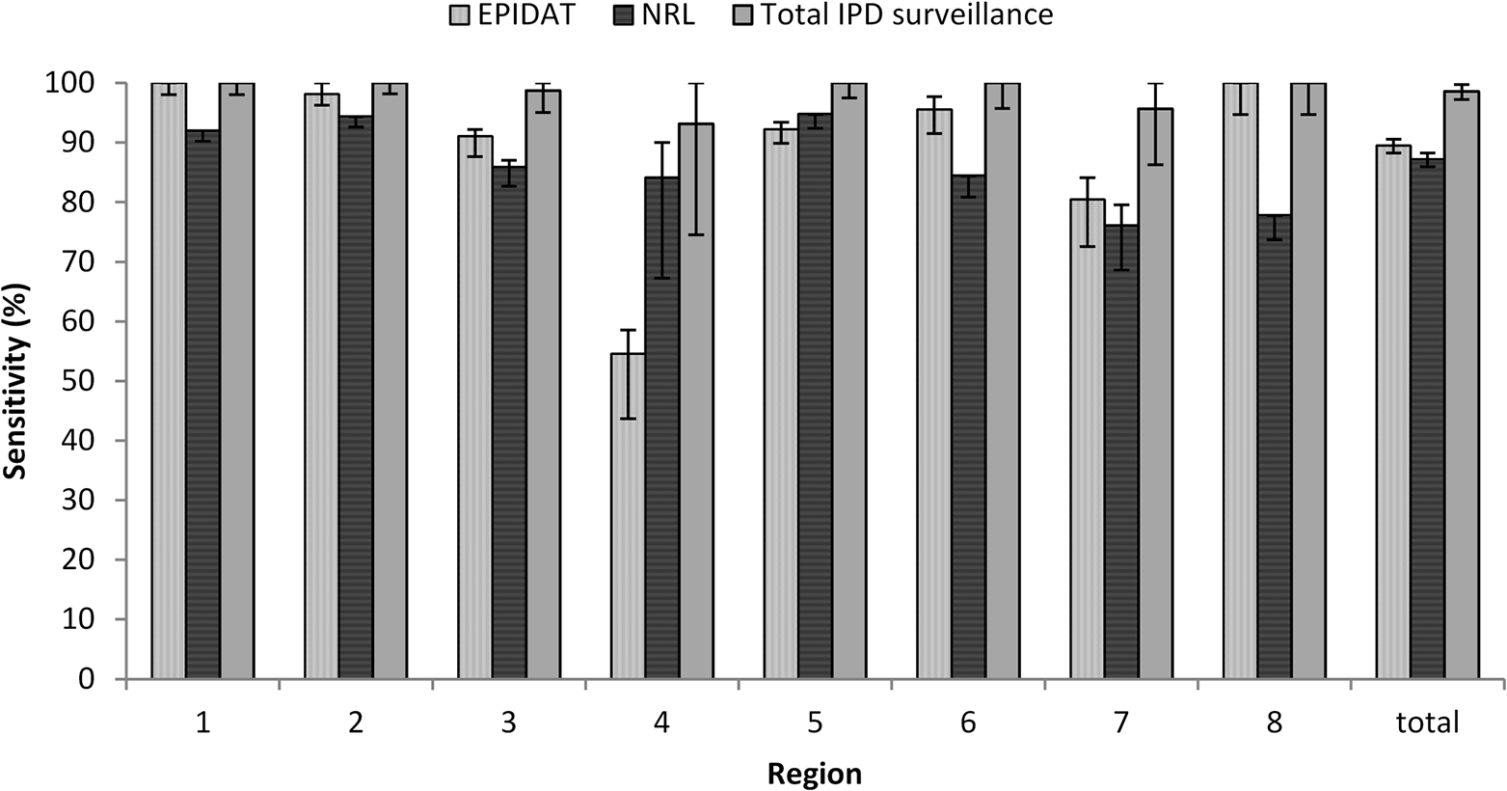
Estimated sensitivity of IPD reporting stratified by region according to the Nomenclature of Units for Territorial Statistics (NUTS 2), 2013, CZ. Error bars reflect 95% confidence intervals.

By geographical region, reporting sensitivity ranged between 92% and 100% for the consolidated IPD surveillance (p<0.001). By source, the estimated sensitivity ranged between 54% and 100% for EPIDAT and between 76% and 94% for NRL in individual regions (Fig 6).

The relative OR for source dependency obtained according to the method of Hook *et al*. (1992) was 0.99, 0.98, 1.06 and 1.03 for the years 2010, 2011, 2012 and 2013, respectively. Estimates for source dependency obtained by the method according to Sekar and Deming (1949) using stratified data by region and age group indicated correlation coefficients off zero in all years and for both stratifying variables. Statistical significance was observed in years 2012 and 2013 for stratification by age group (Table 1).

**Table 1:** Estimation of source dependency according to Sekar and Deming [18]. Correlation coefficients and p-values are indicated for the years 2010 to 2013, using data stratified by region and age groups.

year	strata	correlation coefficient	p-value
**2010**	regions	0.39	0.341
	age groups	0.47	0.430
**2011**	regions	-0.35	0.390
	age groups	-0.73	0.160
**2012**	regions	0.49	0.215
	age groups	0.90	0.035
**2013**	regions	0.34	0.413
	age groups	0.88	0.047

## Discussion

In relation to the introduction of PCV vaccination in the CZ, a new surveillance system for IPD was implemented in 2008. In this study we describe its structure and performance for the first time and thereby follow the previous report adressing the time period from 1997 to 2006 [7]. Furthermore we evaluated the reporting sensitivity between 2010 and 2013 and considered these estimates in the discussion of the IPD epidemiological trend in the post-vaccination era.

The overall IPD incidence in the CZ was relatively stable between 2008 and 2012 and was lower than the 2011 European notification rate of 5.6/100.000 [9]. The IPD incidence among children under five decreased drastically from 2008 to 2009, which reflected the probable impact of private PCV vaccination. From 2010 to 2011, the overall IPD incidence increased slightly, whereas the incidence in the <5y age group decreased, reflecting the probable effect of the national IPD childhood vaccination program implemented in 2010. This effect may have been diminished by the increasing sensitivity of reporting during that time and therefore may not be evident in the total incidence. Both, the total incidence and the incidence in 0-4y age groups decreased from 2011 to 2012 and increased again in 2013. During this time, the sensitivity of IPD surveillance remained stable between 96% and 99% and no further significant change in the surveillance system occurred, indicating a real change in the IPD incidence. Equally, the increasing incidence in 2013 cannot be explained by PCR introduction in the NRL, as the number of cases identified by PCR only in the NRL was still very low in 2013 (Six cases, thereof two in children <5y). If the 2013 increase is a real trend or a temporal fluctuation, this has to be monitored in order to evaluate the current vaccination strategy. A detailed vaccine impact analysis including data on vaccine coverage, vaccination status of IPD cases and details on individual serotypes is therefore required.

The estimated sensitivity of reporting for the individual sources as well as for the consolidated IPD surveillance increased significantly over the four year period, reaching a maximum of 99% in 2013. The largest increase in reporting sensitivity occurred from 2010 to 2011 due to the introduction of report reminders, which positively influenced reporting to EPIDAT and the overall data quality.

We estimated the true number of IPD cases in the population by capture-recapture methodology as the basis for the sensitivity calculations. Capture-recapture has some limitations and its use for estimating reporting sensitivity has been discussed critically [12,13]. A prerequisite of this method is the equality of capture probability for all cases and the independency of data sources.

The Czech IPD surveillance system is based on two reporting sources and is organised at the National reference laboratory for streptococcal infections, which is responsible for the distribution of report reminders since 2011 and the reconciliation of the NRL and EPIDAT databases. Since 2011, in particular, we can assume positive source dependency. In order to estimate the extend of source dependency we applied two different mathematical models. Whilst the estimation of source dependency according to Hook *et al* (1992) indicated no essential dependency, the results obtained by calculations according to Sekar and Deming (1949) suggested a certain range of dependency, with statistical significance in years 2012 and 2013 for stratification by age group. Positive source dependency leads to underestimation of the true number of cases in the population and thus to overestimation of the sensitivity [12,13]. The reported sensitivity values in our study should therefore be interpreted as the upper limit of reporting sensitivity. Despite this limitation, our results clearly reflect a positive development of IPD surveillance within the last four years and a stable reporting sensitivity since 2011. Furthermore, sensitivity estimates–even if generated under suboptimal conditions—provide possibilities to adjust reported incidence rates as a basis for correct disease monitoring and vaccination impact assessments [5,12,19–22].

The prerequisit of capture equality was also not completely given in our case as laboratory procedures influenced the reporting to individual sources especially before 2013. In order to adjust for other possible reasons leading to inequality in reporting probabilty, we stratified surveillance data from 2013. This analysis demonstrated variability in reporting sensitivity especially according to region and particularly revealed reporting difficulties in one region. Furthermore, we observed differences in sensitivity of reporting between both data sources, with a slightly better performance of the EPIDAT system in most of the regions. Nevertheless, sensitivity of the consolidated surveillance was > 90% in all strata.

Inequalities in IPD reporting can be explained by several reasons: firstly, IPD is not clearly associated with a single clinical picture and reporting to EPIDAT occurs via different ICD codes. The compliance with the correct codes used for case ascertainment is crucial for the achievement of high reporting sensitivity within the EPIDAT system. Secondly, differences in medical practices or diagnostic procedures can influence detection rates and might lead to underestimation of IPD cases [5]. Other factors might be economic, quality of staff training, unawareness of the importance of reporting or a low level of communication between health authorities [23]. Since details of diagnostic, medical and reporting practices are difficult to determine in the field, the specific reasons for the regional differences in sensitivity of reporting remain a matter of speculation and require further investigation.

## Conclusion

This study illustrates the positive development and stability of IPD surveillance in the CZ within the last four years and is a first attempt to provide adjusted surveillance data. It also describes the use of capture-recapture analyses for sensitivity estimations to monitor corrected epidemiological trends and to assist in estimating developments of surveillance systems. Capture-recapture analyses using stratified surveillance data were beneficial for the detection of individual subgroups with poor reporting sensitivity. Diverse sensitivity of reporting was apparent by region and source (laboratory and epidemiological reporting), giving the possibility of targeted recommendations towards reporting enhancement. Furthermore, this study demonstrates the importance of report reminders as a suportive element and of the close communication between different health authorities in relation to reporting sensitivity.

On the basis of the presented results we recommend the continuation of close cooperation and communication between health authorities in order to enhance reporting sensitivity. Further studies aimed at investigating diagnostic, medical and reporting practices performed in the field are desirable to identify possible reasons for regional reporting differences. The increasing IPD incidence in 2013 should be monitored carefully and a comprehensive vaccine impact analysis is required to assist future decisions on vaccination strategies.

## References

[1] Donkor ES (2013) Understanding the pneumococcus: transmission and evolution. Front Cell Infect Microbiol 3: 7 10.3389/fcimb.2013.00007 23471303 PMC3590460

[2] Randle E, Ninis N, Inwald D (2011) Invasive pneumococcal disease. Arch Dis Child Educ Pract Ed 96: 183–190. 10.1136/adc.2010.191718 21555595

[3] ECDC (2012) Surveillance of invasive pneumococcal disease in Europe, 2010. European Centre for Disease Prevention and Control.

[4] Song JY, Nahm MH, Moseley MA (2013) Clinical implications of pneumococcal serotypes: invasive disease potential, clinical presentations, and antibiotic resistance. J Korean Med Sci 28: 4–15. 10.3346/jkms.2013.28.1.4 23341706 PMC3546102

[5] Hanquet G, Perrocheau A, Kissling E, Bruhl DL, Tarrago D, Stuart J, et al (2010) Surveillance of invasive pneumococcal disease in 30 EU countries: Towards a European system? Vaccine 28: 3920–3928. 10.1016/j.vaccine.2010.03.069 20394721

[6] Isaacman DJ, McIntosh ED, Reinert RR (2010) Burden of invasive pneumococcal disease and serotype distribution among Streptococcus pneumoniae isolates in young children in Europe: impact of the 7-valent pneumococcal conjugate vaccine and considerations for future conjugate vaccines. Int J Infect Dis 14: e197–209. 10.1016/j.ijid.2009.05.010 19700359

[7] Motlova J, Benes C, Kriz P (2009) Incidence of invasive pneumococcal disease in the Czech Republic and serotype coverage by vaccines, 1997–2006. Epidemiol Infect 137: 562–569. 10.1017/S0950268808001301 18796171

[8] Pebody RG, Hellenbrand W, D'Ancona F, Ruutu P (2006) Pneumococcal disease surveillance in Europe. Euro Surveill 11: 171–178. 17075159

[9] ECDC (2013) Surveillance of invasive bacterial diseases in Europe, 2011. European Centre for Disease Prevention and Control

[10] Hanquet G, Lernout T, Vergison A, Verhaegen J, Kissling E, Tuerlinckx D, et al (2011) Impact of conjugate 7-valent vaccination in Belgium: addressing methodological challenges. Vaccine 29: 2856–2864. 10.1016/j.vaccine.2011.02.016 21342667

[11] Savulescu C, Hanquet G, SpIDnet-group. Impact of higher-valency conjugate vaccines on invasive pneumococcal disease: preliminary results of a European multicentre project; 2014; Dublin, Ireland.

[12] Brenner H (1995) Use and limitations of the capture-recapture method in disease monitoring with two dependent sources. Epidemiology 6: 42–48. 7888444 10.1097/00001648-199501000-00009

[13] Tilling K (2001) Capture-recapture methods—useful or misleading? Int J Epidemiol 30: 12–14. 11171841 10.1093/ije/30.1.12

[14] Hook EB, Regal RR (1992) The value of capture-recapture methods even for apparent exhaustive surveys. The need for adjustment for source of ascertainment intersection in attempted complete prevalence studies. Am J Epidemiol 135: 1060–1067. 1534441 10.1093/oxfordjournals.aje.a116400

[15] Tilling K, Sterne JAC (1999) Capture-recapture models including covariate effects. Am J Epidemiol 149: 392–400. 10025483 10.1093/oxfordjournals.aje.a009825

[16] Farrington CP (2002) Interval estimation for Poisson capture-recapture models in epidemiology. Stat Med 21: 3079–3092. 12369083 10.1002/sim.1223

[17] R_Core_Team (2013) R: A language and Environment for Statistical Computing.

[18] Sekar CC, Deming WE (1949) On a Method of Estimating Birth and Death Rates and the Extent of Registration. Journal of the American Statistical Association 44: 101–115.

[19] German RR, Lee LM, Horan JM, Milstein RL, Pertowski CA, Waller MN, et al (2001) Updated guidelines for evaluating public health surveillance systems: recommendations from the Guidelines Working Group. MMWR Recomm Rep 50: 1–35; quiz CE31-37. 18634202

[20] Gjini A, Stuart JM, George RC, Nichols T, Heyderman RS (2004) Capture-recapture analysis and pneumococcal meningitis estimates in England. Emerg Infect Dis 10: 87–93. 15078602 10.3201/eid1001.030123

[21] Howitz MF, Samuelsson S, Molbak K (2008) Declining incidence of meningococcal disease in Denmark, confirmed by a capture-recapture analysis for 1994 and 2002. Epidemiol Infect 136: 1088–1095. 17892628 10.1017/S0950268807009466 PMC2870894

[22] Schrauder A, Claus H, Elias J, Vogel U, Haas W, Hellenbrand W (2007) Capture-recapture analysis to estimate the incidence of invasive meningococcal disease in Germany, 2003. Epidemiol Infect 135: 657–664. 16938141 10.1017/S0950268806007151 PMC2870604

[23] Declich S, Carter AO (1994) Public health surveillance: historical origins, methods and evaluation. Bull World Health Organ 72: 285–304. 8205649 PMC2486528

